# Innovative Materials in Italy for Eco-Friendly and Sustainable Buildings

**DOI:** 10.3390/ma14082048

**Published:** 2021-04-19

**Authors:** Francesco Colangelo, Ilenia Farina, Marta Travaglioni, Cinzia Salzano, Raffaele Cioffi, Antonella Petrillo

**Affiliations:** Department of Engineering, University of Naples Parthenope, Centro Direzionale, Is. C4, 80143 Naples, Italy; francesco.colangelo@uniparthenope.it (F.C.); ilenia.farina@uniparthenope.it (I.F.); marta.travaglioni@uniparthenope.it (M.T.); cinzia.salzano@uniparthenope.it (C.S.); raffaele.cioffi@uniparthenope.it (R.C.)

**Keywords:** green concrete, innovative concrete, civil buildings, recycled aggregates, industrial waste

## Abstract

In the last 20 years, there have been a series of seismic events in Italy that have caused serious damage to civil and building structures. This has led to a significant increase in the use of concrete for the reconstruction of new structures and the repair of existing structures damaged by earthquakes. At the same time, the concrete industry is responsible for the most significant environmental damage during the life cycle of the built environment. The environmental disadvantages characterizing the concrete industry are related to the constant growth of the exploitation of natural aggregates. Therefore, it is necessary to use alternative and innovative aggregates that provide good concrete performance and lower environmental impacts. In this study, a very promising route from an environmental point of view is given by the use of artificial aggregates from industrial waste as substitutes for natural aggregates. An innovative low cost and energy saving granulation process has been employed to produce lightweight aggregates using fly ash from the incineration of municipal solid waste and ground granulated blast furnace slag. The final aim of this research is to demonstrate the environmental sustainability of artificial aggregates, through a comparison of three different mixtures.

## 1. Introduction

Italy is one of the countries with the greatest seismic risk in the Mediterranean, due to the frequency of earthquakes that have historically affected its territory and the intensity that some of them have reached, resulting in a significant social and economic impact [1,2]. Earthquakes and telluric phenomena are frequent and have affected various regions from North to South. Emilia Romagna, L’Aquila, and all of central Italy are just some of the locations of the most recent earthquakes, which unfortunately have left collapsed structures on the ground, swept away entire villages, and caused a dramatic loss in terms of human lives [3,4]. Moreover, earthquakes have caused substantial economic damage, estimated for the last forty years at around 135 billion euros, which were used for the restoration and post-event reconstruction [5]. To this must be added the consequences for the historical, artistic, and monumental heritage [6].

In Italy, the ratio between the damage produced by earthquakes and the energy released during the events is much higher than that which normally occurs in other countries with high seismicity, such as California or Japan [7]. For example, the 1997 earthquake in Umbria and Marche produced a picture of damage with an economic damage of about 10 billion euros comparable to that of California in 1989 with 14.5 billion US $, even though it was characterized by about 30 times lower energy [8]. This is mainly due to the high population density and the considerable fragility of our building stock.

The most recent regulations have acknowledged the presence of a widespread seismic hazard that does not spare areas that the previous classifications declared free from a probability of seismic events of any significance. This circumstance has accentuated the presence on the Italian territory of buildings, even quite recent ones, which although built according to the law, do not meet the requirements of seismic zones [9].

For this reason, the need to build buildings resistant to seismic phenomena—or to adapt existing ones by improving their resistance—is spreading more and more, to prevent damage caused by the earthquake. In this regard, over the past twenty years, earthquake science and in particular seismic engineering have undergone a technical-scientific transformation with respect to earthquake resistance and the first seismic regulations for the protection of new buildings [10]. The first innovative aspect concerns the buildings and lies in the recognition of the importance of the seismic protection of the existing heritage. Especially in Italian towns, but also in Europe, the existing built heritage, even the minor one, is in many cases of great historical and cultural importance and fits into environmental contexts that are also of considerable value [11]. It is therefore necessary, also for reasons of historical continuity, to protect this heritage in its entirety, including monuments, public and private buildings, productive activities, infrastructures, and landscape resources [12,13]. However, safeguarding an artistic, cultural, environmental heritage, often very vulnerable to seismic action, and concerning sometimes densely populated areas, not only poses totally new engineering problems, it requires legislative and implementation measures to safeguard on a large scale, which require research, investigations, controls on the physical and built environment [14]. Over the last decade, a great effort and several challenges have been faced by researchers and civil engineers to investigate effective innovative and reinforcement techniques for existing structures.

From the physiological degradation of structures to destructive seismic events, the building heritage requires adequate analysis and planning for maintenance actions, not only of a demolition and reconstructive nature, but also, and above all, for the safeguarding of the existing one that has specific or limited problems, even if demanding from the point of view of materials and future durability [15]. Conventional materials like cement and technologies used in construction have numerous advantages, including the relatively low cost of raw materials, although the presence of cement in the mixture, and, in particular, the presence of clinker, makes the concrete a high environmental impact material [16]. They are also suitable for many construction applications, but often unsuitable for some applications, and they can undergo rapid deterioration [17].

Very often, however, the retrofit and rehabilitation of existing structures with conventional materials is not possible, and therefore, demolition with consequent reconstruction is used [18]. In these cases, it is necessary to use new materials and technologies in order to increase the durability of the structures [19,20].

A potential use in civil infrastructures for the restoration of existing structures and also for new structures is represented by fiber-reinforced polymer matrix (FRP) composites [21,22]. One of the advantages of using FRP composites as building materials comes from the ability to customize these materials by combining the fibers with the polymer resin matrix [23]. In these materials, the fibers carry the load in predetermined directions, and the resin transfers the stresses between the closest fibers, thanks to the adhesion, and also gives some protection to the fibers [24]. Such behavior offers the designer a wide range of materials to meet the specific requirements, and the great opportunity to reinforce conventional materials used for civil buildings [25]. Moreover, compared to metals, composites are not subjected to rusting, and it makes them attractive in applications where corrosion occurs, such as composite bars and grids, as well as cables for pre- and post-tensioning and use in cable holders [26]. Thanks to their good resistance to corrosion and solvents, composite materials need less maintenance than traditional materials, and it results in lower overall life cycle costs [27]. Another technique for securing and improving for seismic adaptation is the installation of fiber optic sensors for structural monitoring of the building. This is an innovative combined system of fiber optic sensors and accelerometers, capable of sending real-time information on the behavior of the building in the event of seismic stress or other catastrophic events [28]. The monitoring network sends alert or alarm signals and allows you to identify the most urgent action to be applied, use visual investigations in the event of an alert, or simply study the data recorded by the sensors in the event of a simple alarm [29].

In order to guarantee greater performance in new and existing structures in terms of thermal and acoustic insulation, research is increasingly oriented towards the use of innovative and eco-compatible building materials and new technologies. In this regard, several studies pointed out the beneficial employment of recycled and waste materials as substitutes for virgin materials to reduce the use of raw materials, soil and the energy-intensive processes involved. In particular, several case-studies include the use of industrial [30], agricultural [31,32], construction and demolition waste [33], recycled aggregates [34,35,36,37] and lightweight aggregates (LWA) [38,39,40,41,42] made of waste materials as good candidates to partially substitute natural aggregates [43].

In this work, cold bonding pelletization process has been used to produce lightweight aggregates, employing fly ash from the incineration of municipal solid waste and ground granulated blast furnace slag. The application of such a process has economic and environmental advantages due to the reduced energy requirement, since it is carried out at room temperature and no gaseous emission is involved. The study also assessed the life cycle environmental impact of lightweight aggregates (LWA) using life cycle assessment (LCA). The entire process includes raw materials and the manufacturing of recycled aggregates, along with the energy consumption amount, in order to assess environmental impacts. Furthermore, the study compares and analyzes the environmental impact of three different mixtures with recycled aggregates.

## 2. Literature Review for Sustainable, Safe, and Resilient Buildings

The Italian building heritage consists of a large number of existing concrete buildings, thanks to the ease of production and the procurement of constituent materials, and also thanks to the good mechanical performance that it guarantees. For this reason, concrete is today the most used building material in the world, and consumption is second only to that of water [44]. Furthermore, the development of a nation is also related to concrete [44], but the constant growth of concrete consumption has significant disadvantages. The presence of cement in its mixture, and in particular of clinker, makes concrete have a high environmental impact. In structures built with conventional concrete, 90% of the energy necessary for their construction is spent in the production phase of the raw materials, especially clinker, while only the remaining 10% relates to the packaging of the concrete, transport and use on site [16]. In this scenario, the need to reconstruct buildings affected by a natural disaster, such as an earthquake, should direct the choice of eco-sustainable materials, able to give the structures the characteristics that comply with current seismic standards and, in general, make the buildings sustainable.

In this respect, the building envelope plays a fundamental role. In particular, there is a tendency to produce insulating materials by exploiting waste recycling, increasingly adhering to circular economy policies. In order to reduce energy consumption, thermal and acoustic insulation plays an important role. A correct thermal and acoustic insulation in fact involves considerable savings in energy and economic terms. In addition to thermal and acoustic efficiency, a correct building envelope can reduce seismic vulnerability of the infill by increasing its strength. The answer to both is the use of lightweight structures. In fact, the main use of lightweight structural concrete is to reduce the load of a concrete structure, which allows a reduction in the size of the structural elements (beam, pillar, and foundation) and a consequent reduction of earthquake forces on the structure [45].

Under the eco-sustainability of alternative and sustainable materials, the literature proposes several studies focused on LCA analysis, to understand the extent of the phenomenon. These studies demonstrate the eco-sustainability of artificial aggregates, some of which involve the use of FA, capable of satisfying the typical functional characteristics of building materials.

Rosado et al. [46] compared the production of natural and mixed recycled aggregates through the LCA methodology. The results show that the environmental sustainability of recycled aggregates is better than that of natural materials in several impact categories, including global warming.

Tam et al. [47] reviewed the literature on the production and use of recycled aggregates in concrete and other civil engineering works from 2000 to 2017. Among the case studies examined, in some of these, the CO_2_ emissions and the embodied energy of recycled aggregates were compared with the virgin aggregates. The results of the comparison show that the recycled aggregates are very promising.

Hossain et al. [48] proposed research on the environmental impacts for the production of recycled aggregates from waste, assessed with LCA methodology. Specifically, a comparison was made between emissions from artificial aggregates and those associated with natural aggregates. The results show that the recycled aggregates produced reduced greenhouse gas emissions by 65%, and allowed a saving of 58% on the consumption of fossil resources.

Kurda et al. [49] compared the impacts of concrete mixes, which contain different incorporation ratios of fly ash and recycled concrete aggregates, with and without superplasticizer. The results regarding global warming suggest that this is mainly affected by the cement content in concrete, and that it decreases significantly with the incorporation of FA. In another work [50], Kurda et al. focused mainly on the effect of high incorporation ratios of FA and recycled concrete aggregates on the carbonation resistance of concrete. The author proposes an alternative method for the mitigation of CO_2_ emissions derived from the carbonation process. The alternative way consists in the sequestration of a high amount of CO_2_ in the concrete, producing concrete with a high rate of carbonation. The research results show that the carbonation of concrete increases up to 3 and 6 times with the incorporation of 30% and 60% of FA, respectively.

Rodríguez-Robles et al. [51] presented a review on the environmental impacts generated by the production and use of recycled aggregates and other common components of concrete. Furthermore, it carried out an LCA analysis which showed the eco-sustainability of artificial aggregates in terms of reduction of greenhouse gas emissions, resource depletion, land use, and waste.

Serres et al. [52] dealt with the environmental aspects concerning the use of alternative aggregates obtained from waste materials. The results of the LCA show that the recycled samples exhibit good environmental behavior, even if the recovered materials (sand and aggregates) involve different operations (crushing, extraction, etc.).

## 3. Materials and Methods

### 3.1. Artificial Lightweight Aggregates

Three different mixtures were prepared in order to manufacture lightweight aggregates through the cold bonding pelletization process. The experimental activity was carried out in the laboratory M.A.T.E.C of the Department of Engineering of the University of Naples “Parthenope”. Portland limestone cement (CEM II/A-L 42.5R), ground granulated blast furnace slag (GGBFS) and municipal solid waste incinerator (MSWI) fly ash were employed as components of the binding systems. The MSWI fly ash employed comes from an incineration plant located in Acerra (Naples, Italy) and their content was 80% by weight in all the mixtures. According to the European Waste Catalogue (2000/532/EC [53]), this waste is listed as hazardous material labeled with the code 19.01.05* [54] and cannot be used or even landfilled without prior treatment. Ground granulated blast furnace slag (GGBFS) is a by-product of steel mills. The content of the main components cement, MSWI fly ash and GGBFS samples has been determined through X-ray fluorescence, carried out at ambient temperature using the BRUKER Explorer S4 spectrophotometer. 

From the result of the fly ash XRF (X-ray fluorescence) analysis, the main component is calcium oxide (CaO) with a percentage amount of about 24%, followed by silica (SiO_2_) with about 3%, alumina (Al_2_O_3_) with about 1.5%, and sodium oxide (Na_2_O) with about 13%. Chlorine is present in significant concentrations (approximately 21%), while sulfur is on average in lower concentrations (approximately 9%). The composition of the limestone cement in terms of equivalent oxides is obtained by firing in suitable ovens mixtures of limestone and clay. The limestone (CaCO_3_) decomposes, providing about 67% of calcium oxide (CaO), the clay decomposes into about 17% of silica (SiO_2_), about 4% of alumina (Al_2_O_3_), and about 3% of iron oxide (Fe_2_O_3_). As for the slag deriving from the production of cast iron and steel, their composition is high in Fe_2_O_3_ (approximately 25%) and in CaO (approximately 17%).

The accurate compositions of FA, GGBFS and CEM II expressed in terms of mass fraction as a percentage by weight (wt%) are given in Table 1.

Stabilization process is not very effective for MSWI fly ash, due to the low immobilizing capacity of a cement matrix towards chlorides and sulfates. For this reason, a preliminary washing step was applied to remove soluble salts as much as possible. Specifically, a two-step pre-treatment wash was submitted to the MSWI fly ash sample (19 kg), with a liquid/solid ratio equal to 2.5:1 and a retention time of 1.5 h for each step. This type of process is complex, as it requires a counter-current operation with two washing steps. However, it requires reduced water consumption, with consequent optimization of the water amount and process economy. Washing operating conditions for pre-treatment were chosen over process optimization tests performed in previous works [55]. In fact, in [55], a preliminary washing treatment was optimized to remove as much soluble salts as possible. For this purpose, two different operating conditions have been developed (single and double phase), from which it emerged that the effectiveness of stabilization increases in the order of single-step washed ash 3:1 and two-step washed ash 2:1 with a retention time of 30 min in deionized water at room temperature [55]. Therefore, the adoption of two-step washing pre-treatments with a higher retention time allowed a better removal of soluble salts even at low L/S (liquid/solid) ratio values. After fly ash washing, a drying process was performed to remove the water from fly ash. The drying process took place at a temperature of 45 °C in an oven for 24 h. Finally, three mixtures were prepared, in which the mass of washed fly ash was kept constant (80%), and the mass of cement and ground granulated blast furnace slag varied. In the three mixtures of lightweight aggregates labeled as LWA A, LWA B and LWA C, 15%, 10% and 5% by weight of GGBS and 5%, 10% and 15% by weight of cement were employed, respectively, as shown in Table 2. 

**Figure 1:**
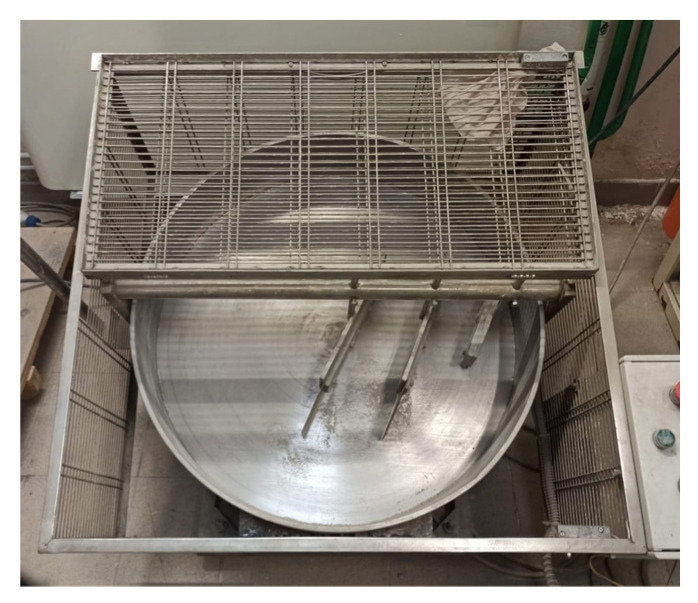
Cold bonding pelletization pilot-scale disk granulator.

Cold bonding pelletization was carried out by means of a pilot-scale system show in Figure 1. The disk granulator is fitted with a rotary and tilting plate (d = 80 cm), of which it is possible to vary the rotary velocity and tilting angle between large limits. In the experiment, the settings for the rotating velocity were set at 45 rpm and the tilting angle at 45°. During the cold bonding pelletization process, FA, GGBFS, and CEM II were subjected to mixing with progressive addition of water (0.99 L in LWA A; 0.11 L in LWA B; 1.04 L in LWA C), capable of forming inter-particle bonds between the fine powders for the formation of particles with larger diameters, which are the LWA. 

**Figure 2:**
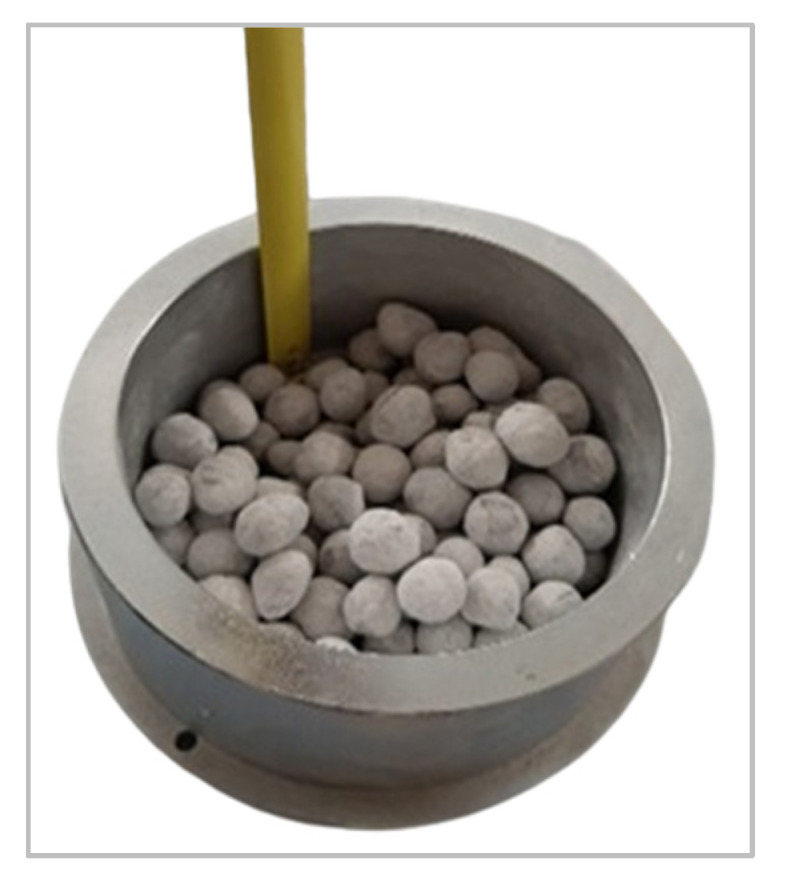
Final artificial lightweight aggregates (LWA).

For each mixture, about 4 kg of aggregates were obtained from a cold bonding pelletization process, as shown in Figure 2.

The aggregates obtained (Figure 2) were cured for 28 days at room temperature and relative humidity of 95% to achieve the suitable mechanical properties. The materials immobilized through the granulation process can be used as substitutes for natural aggregates, whose environmental benefits will be demonstrated by the following LCA study.

### 3.2. Life Cycle Assessment

One of the basic instruments for adopting an Integrated Product Policy is the Life Cycle Assessment (LCA). The importance of this technique lies primarily in its methodology, which consists of the evaluation as linked and dependent of all phases of the production process. LCA has assumed a leading position among the tools produced for the study of industrial systems and is expanding rapidly nationally and internationally. At the European level, the strategic importance of LCA adoption as a fundamental and scientifically relevant tool for defining important environmental aspects is clearly reflected in the Green Papers COM 2001/68/EC [56] and COM 2003/302/EC [57] on the Integrated Product Policy [58] and, at least indirectly, in the European Regulations EMAS (Reg. 1221/2009 [59]) and Ecolabel (Reg. 1221/2009 [60]), respectively. The methodology for the LCA study is organized and standardized by the UNI EN ISO 14040 family of standards, in particular UNI EN ISO 14040: 2006 [61] and UNI EN ISO 14044:2006 [62]. According to the standards, the LCA studies include four phases: the description of the goal and scope, the Life Cycle Inventory (LCI) analysis, the Life Cycle Impact Assessment (LCIA) and the interpretation of LCIA. In this LCA study, the methodology adopted based on the evaluation of key problems using ReCiPe 2016 midpoint (H). Indicators at the midpoint level are specified at the intermediate level along the mechanism, and the hierarchical perspective is considered the default method, since it performs an impact evaluation in the medium term.

## 4. Analysis for Optimizing Sustainability of LWA

### 4.1. Goal and Scope Definition

**Figure 3:**
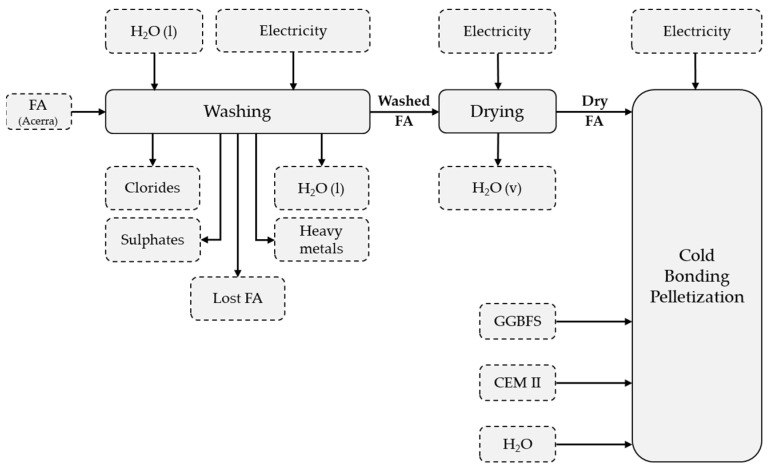
LWA production process flow chart.

The aim of this work is to apply the LCA methodology to the production processes of LWA. The objective of the study is to assess the environmental load associated with the production of LWA. The comparative study, or a comparison of the environmental performance from the three mixtures described in Section 3.1, shall be carried out. Figure 3 shows a schematization of the production process of LWA, in which the sequence of the operations is reported: washing, drying, and cold bonding pelletization (CBP). For each represented process, the respective inputs and outputs are also reported, as shown in Figure 3.

In order to produce lightweight aggregates LWA A, LWA B and LWA C, three cold bonding pelletization processes CBP A, CBP B and CBP C were performed.

**Figure 4:**
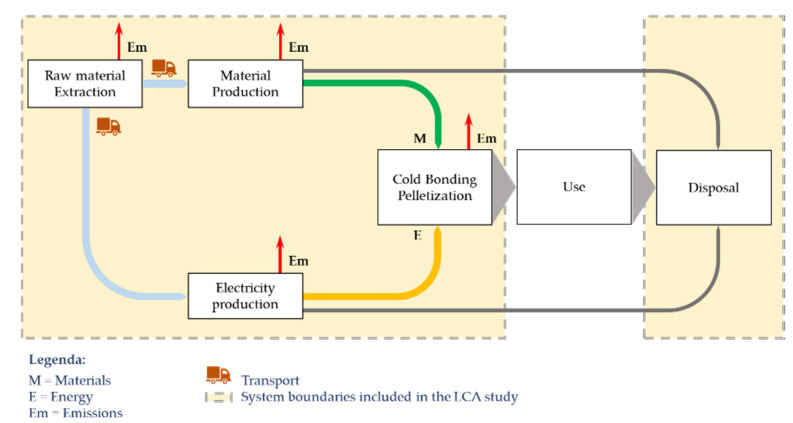
Representation of system boundaries.

According to other LCA studies about artificial aggregates [63], the functional unit for the life cycle impact assessment of LWA was 1 kg to facilitate the management and application of the data. The analysis was carried out according to a “cradle-to-gate” approach. A cradle-to-gate system boundary limits the scope of the processes, the emissions and energy studied in the LCA until the production stage, excluding the use and end-of-life stages. Therefore, the production stages of raw materials, materials, transport of raw materials, production of aggregates and disposal of materials were included, as shown in Figure 4. 

The wastewater disposal phase resulting from the washing of the MSWI FA has been excluded. In particular, the contaminated water is collected in special containers, so that it can be sent to external water treatment plants.

### 4.2. Life Cycle Inventory (LCI)

Life cycle inventory analysis provides for the collection of data relating to all inputs and outputs (Figure 3). Data were obtained from laboratory analyses, datasets, and literature. The material production processes available in the databases concern cement (CEM II/A-L 42.5R), deionized water and electricity. The data concerning the production of MSWI FA are of primary type since they are reconstructed starting from the Environmental Declaration 2020 issued by RINA S.p.A. for A2A Ambiente [54]. The mass allocation for FA is equal to 1.5% (0.015 kg/MJ). GGBFS data are obtained from the literature. It is produced downstream of the steel production process. With reference to Van den Heede et al. [64], the quantity produced is 0.24 kg per kg of steel. For this reason, the mass allocation coefficient is 24%.

### 4.3. Results of Life Cycle Impact Assessment (LCIA)

In this section, the impacts deriving from the single granulation process are evaluated using the Recipe 2016 Midpoint (H) method. Recipe 2016 Midpoint (H) uses an environmental mechanism as a basis for modeling [65]. An environmental mechanism can be seen as the series of effects on human health, ecosystems and available resources. For example, for climate change, it is known that a certain number of substances increase the “radiative forcing”, or the phenomenon according to which heat cannot radiate from the Earth to space, so the more energy is trapped on the Earth, the more the temperature rises. Consequently, changes in the habitats of living organisms can be expected, leading to the eventual extinction of living species [65].

The LCI results were characterized according to ReCiPe 2016 Midpoint (H) method, and subsequently normalized according to the ReCiPe 2016 v1.1 (H), Midpoint Normalization, World, excl biogenic carbon (person equivalents) method.

In fact, the characterization allows the calculation of the impact category indicator results. The outcomes are the potential impacts for each of the categories considered, along with their unit of measurement. Therefore, they cannot be compared with each other.

**Figure 5:**
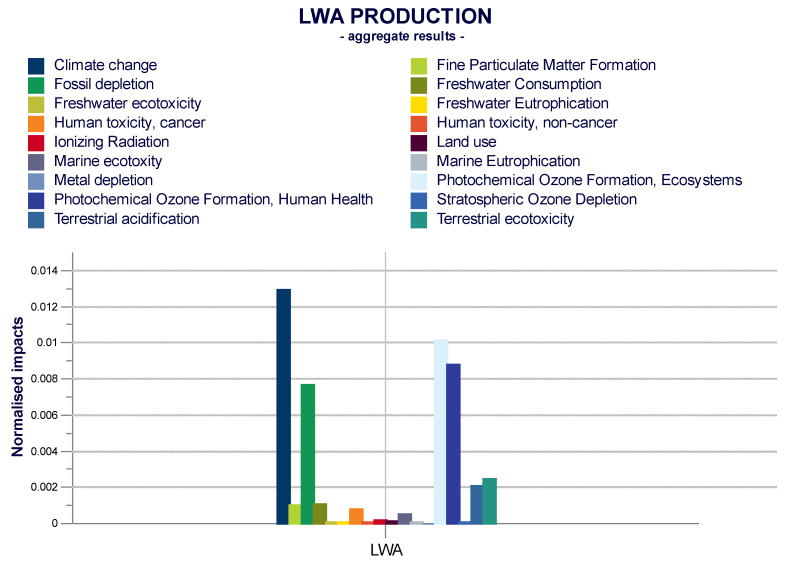
Normalized results by ReCiPe 2016 v1.1 (H), Midpoint Normalization, Word Excel, biogenic carbon (person equivalents).

In order to compare the results, the normalization was carried out. In this phase, the potential impacts relating to each of the categories considered, obtained from the characterization phase, are related to normalization factors, so as to be expressed with the same reference unit. As shown in Figure 5, the climate change category is the most impactful compared to the production of LWA, which includes the process of washing, drying and cold bonding pelletization (CBP).

The climate change category looks at release of CO_2_ emissions into the atmosphere. Carbon dioxide is a climate-altering compound that accentuates the greenhouse effect. In turn, the greenhouse effect causes imbalances in the air, water, and biological sector, with prospectively increasingly significant consequences for man and the terrestrial ecosystem. For these reasons, CO_2_ production is one of the key issues and is the most interesting and debated category from an environmental point of view. The midpoint characterization factor for climate change is global warming potential (GWP). The GWP expresses the amount of additional radiative forcing integrated over time caused by the emission of 1 kg of (greenhouse gases—GHG) relative to the integrated additional radiative forcing in the same time horizon caused by the release of 1 kg of CO_2_. The midpoint characterization factor of any GHG and any time horizon produces a specific GWP with the unit kg CO_2_ eq. (equivalent).

**Figure 6:**
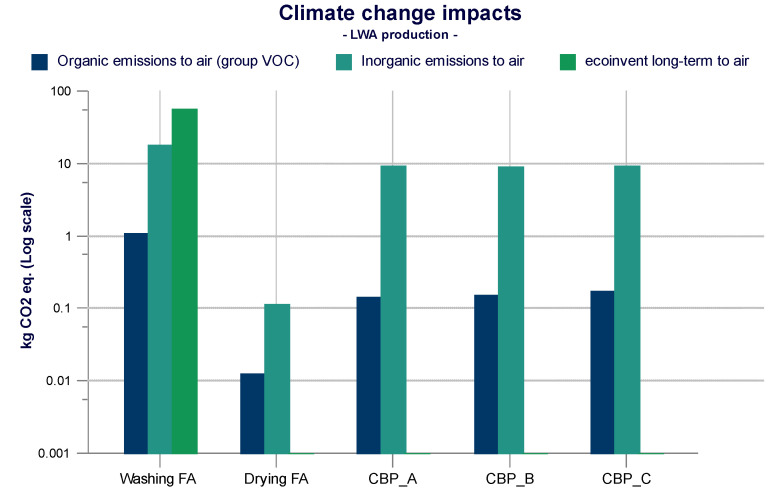
Emission to air for climate change of the LWA process.

By analyzing the climate change category referred to all processes included for the production of LWA (washing—drying—cold bonding pelletization), the impacts mainly derive from inorganic and organic air emissions, as shown in Figure 6.

From the observation of Figure 6, the washing process causes considerably greater impacts than all the other processes included in the analysis. This result is due to the mass allocation of fly ash equal to 1.5% (0.015 kg/MJ). The whole production process of LWA is responsible for the production of 104 kg CO_2_ eq. which is mainly responsible for the washing process with about 76 kg CO_2_ eq., drying 0.13 kg CO_2_ eq., CBP A 9.6 kg CO_2_ eq., CBP B 9.3 kg CO_2_ eq., CBP C 9.3 kg CO_2_ eq. Total inorganic emissions are equal to 46.15 kg CO_2_ eq., and they mainly concern carbon dioxide (including fossil). Carbon dioxide emissions are mainly related to washing with about 18 kg CO_2_ eq. Following, CBP A with 9.4 kg CO_2_ eq., CBP C with 9.2 kg CO_2_ eq., CBP B with 9.1 kg CO_2_ eq., and finally drying with 0.11 kg CO_2_ eq.

Organic emissions refer to the VOC group and concern methane. A total of 1.57 kg CO_2_ eq., the pre-treatment of fly ash is the most impactful process, with 1.08 kg CO_2_ eq. Drying related emissions are negligible (0.014 kg CO_2_ eq.), while for other processes, the methane emitted is 0.177 kg CO_2_ eq. for CBP A, 0.157 kg CO_2_ eq. for CBP B, and 0.145 kg CO_2_ eq. for CBP C.

**Figure 7:**
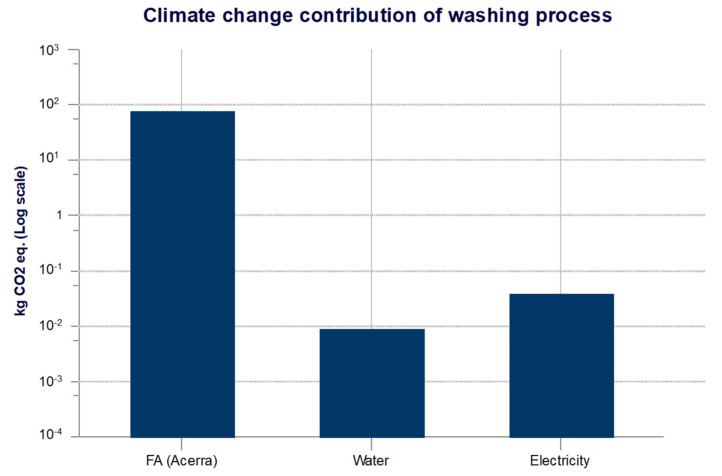
Components contribution of washing process.

The washing process is also responsible for long-term emissions to air. The ReCiPe Midpoint (H) method includes a 100-year perspective, but also includes issues over a longer time horizon. In fact, an emission is classified as “long-term” if it is released into the environment more than 100 years after the activities considered in the life cycle have taken place. Therefore, the decisive factor for the “long-term” classification is the moment in which an emission is released into the environment, and not the moment in which it causes its impact. Long-term emissions to air include only carbon dioxide, with emissions equal to 56 kg CO_2_ eq. Since the washing process is overall more impactful, the emissions to air in terms of CO_2_ eq. from FA, water and electricity respectively are analyzed in Figure 7.

**Figure 8:**
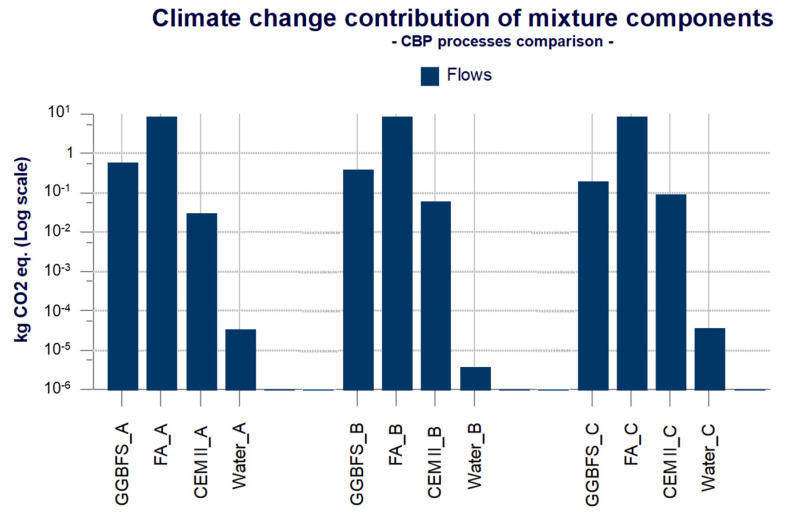
Components contribution of cold bonding pelletization processes.

The washing process is responsible for about 76 kg CO_2_ eq. of which fly ash with about 75.6 kg CO_2_ eq. are mainly responsible; the values of water (0.009 kg CO_2_ eq.) and electricity (0.04 kg CO_2_ eq.) are negligible compared to the total. By analyzing the cold bonding pelletization processes, the contributions of the components of the mixtures LWA A, LWA B, LWA C are reported in Figure 8.

**Figure 9:**
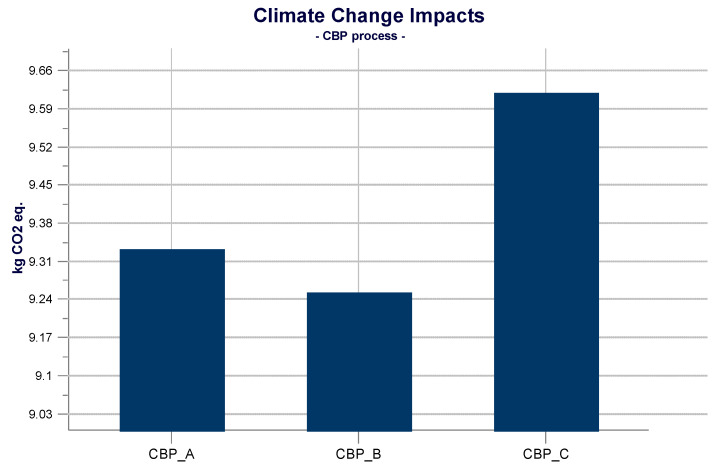
Comparison of the impacts of cold bonding pelletization (CBP) processes.

In the graph (Figure 8), the energy component has been excluded, as the amount of electricity to feed the granulator (0.08 kg CO_2_ eq.) is negligible. Furthermore, the electricity is the same for the three cold bonding pelletization processes, providing the same environmental contribution, making the comparison not useful. From Figure 8, it is possible to notice that cold bonding pelletization impacts derive mainly from the contributions of the GGBFS and FA respectively with 0.6 kg CO_2_ eq., and 9 kg CO_2_ eq. about CBP A. While for CBP B and CBP C, the contributions of GGBFS and FA are respectively 0.4 kg CO_2_ eq., and 8.7 kg CO_2_ eq., for CBP B, and 0.2 kg CO_2_ eq., and 9 kg CO_2_ eq. for CBP C. From this, it follows that overall, the process of cold bonding pelletization less impactful is the CBP B, as shown in Figure 9.

As previously described, LWA production consists of three processes, respectively washing, drying, and cold bonding pelletization (CBP). The differences among the production of LWA A, LWA B and LWA C are the processes of CBP A, CBP B, and CBP C, as the washing and drying processes are unique for all LWA. Therefore, washing and drying contribute with the same impacts unlike cold bonding pelletization. From the results obtained from the LCA analysis (Figure 9) it can be deduced that since CBP B is the less impactful cold bonding pelletization process, LWA B is also less impactful than LWA A and LWA C.

### 4.4. Discussion of Interpretation of LCIA

In this paragraph, the results of LCIA are interpreted. The interpretation of the results is based on the results obtained in the previous phase. In this section, the results are discussed, identifying the key aspects to make decisions to improve the model, in accordance with the goal and scope of the study. The different mix design of the three mixtures involves different contributions related to the components and compared to cold bonding pelletization processes. The most impactful cold bonding pelletization process is CBP A, even though the values of the three processes are very close to each other. The reason is due to the mix design, in particular to the greater presence of GGBFS (15%) and lower presence of cement (5%). In fact, the amount of FA remains constant, while the amounts of GGBFS and CEM II changes. One kilogram of GGBFS has an impact of 4.27 kg CO_2_ eq./kg while 1 kg of CEM II has an impact of 0.68 kg CO_2_ eq./kg. One might think, therefore, that mixture C has less impact, since the amount of CEM II is maximum (15%) and that of GGBFS is minimum (5%), but this does not occur. Indeed, in mixture C, the impacts of CEM II are greater and those of GGBFS are less, but they are compromised by the impacts of water. For mixture A and mixture C, greater impacts related to water are recorded (3.6 × 10^−5^ kg CO_2_ eq.), although they are still lower than those of the components. Conversely, in mixture B, it is verified that the impacts of GGBFS are lower than in mixture A and higher than in mixture C. The impacts of cement are higher than in mixture A and lower than in mixture C. In light of these considerations, it can be said that the key aspects of the process are the main constituents of LWA (FA, GGBFS, CEM II) and, above all, the mix design.

To better understand the differences between the contributions of the constituents of the mixtures, the percentage contribution for each of them is shown in Table 3.

However, as described above, water affects the overall impacts resulting from the cold bonding pelletization processes. Although the amount of water is very low in all mixtures, its impact is not negligible. In particular, mixture B for the production of LWA B requires a minimum amount of water (0.11 L) if compared with 0.99 L for the production of LWA A and 1.04 L for the production of LWA C. This determines a significant difference in environmental impacts among the three mixtures. In mixture B, the impacts are less than one order of magnitude compared to the other two mixtures. In fact, the impacts of water in the cold bonding pelletization process for LWA B amount to 3.83 × 10^−6^ kg CO_2_ eq., while for LWA A and LWA C, the impacts have a value of 3.52 × 10^−5^ kg CO_2_ eq. and 3.72 × 10^−5^ kg CO_2_ eq., respectively. Therefore, water also plays a key role in the process.

**Figure 10:**
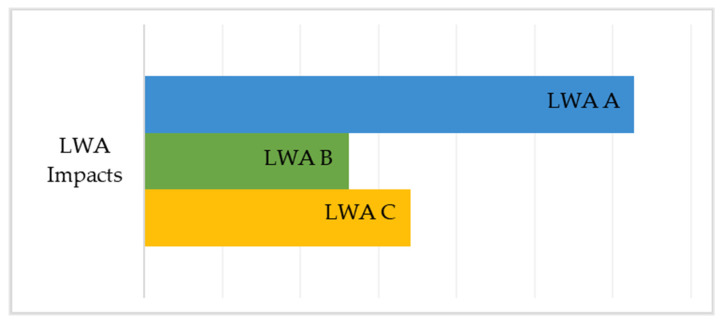
Comparison of the total impacts of LWA mixtures in cold bonding pelletization process.

Considering the key aspects, it appears that the cold bonding pelletization process of mixture B turns out to be the optimal process among the three. Overall, including all processes (washing, drying and cold bonding pelletization), LWA B are the least impactful aggregates with 34.6 kg CO_2_ eq., compared to LWA A (35 kg CO_2_ eq.) and LWA C (34.7 kg CO_2_ eq.), as shown in Figure 10.

Therefore, when choosing the best mixture from an environmental point of view, it is necessary to consider not only the quantities of precursors (FA, GGBFS, CEM II), but also the water needed for the LWA formation process.

## 5. Conclusions

Recently, population growth, urbanization and the economic expansion of industrialized countries have led to the uncontrolled exploitation of natural resources. Therefore, the use of secondary raw materials is encouraged, and the emergence and development of a market for recycled and recovered materials is favored. The proposed LCA study is in line with the objective of producing and calculating the environmental impacts of recycled aggregates in order to preserve natural resources and confirm their eco-sustainability.

Observing the ISO 14,040 and ISO 14,044 standards, this study assessed the environmental impact of the life cycle of lightweight artificial aggregates with a functional unit equal to 1 kg, and a cradle-to-gate approach. The LCA analysis of the whole process has shown that the process with the greatest impact is the pre-treatment process of FA (76 kg CO_2_ eq./kg), which are classified as hazardous waste, due to the high content of heavy metals, chlorides and sulfates. Therefore, FA pose a threat to the environment and to the safety of exposed organisms. In fact, the washing process alone does not produce significant impacts, despite the use of water, as the impacts derive exclusively from the use of FA (75.6 kg CO_2_ eq./kg), industrial by-products which must necessarily be stabilized, regardless of their intended use.

The drying process following washing amount to 0.13 kg CO_2_ eq/kg.

The differences in the production of LWA A, LWA B and LWA C are the processes of CBP A, CBP B and CBP C, as the washing and drying processes are unique for all LWA. The impacts resulting from the CBP A, CBP B and CBP C process are respectively 9.63 kg CO_2_ eq./kg, 9.26 kg CO_2_ eq./kg, and 9.34 kg CO_2_ eq./kg. From the results obtained through the LCA analysis, it emerges that CBP B is the least impacting cold bonding pelletization process, and consequently it can be stated that the LWA B system is less impactful than LWA A and LWA C; in fact, the impacts of LWA A, LWA B and LWA C are respectively 35 kg CO_2_ eq./kg, 34.6 kg CO_2_ eq./kg, and 34.7 kg CO_2_ eq./kg.

The production process of LWA includes washing, drying and cold bonding pelletization (CBP) process. The washing process is responsible for 76 kg CO_2_ eq./kg, drying for 0.13 kg CO_2_ eq./kg, and cold bonding pelletization for about 9.4 kg CO_2_ eq./kg. The energy component has been excluded from washing and CBP processes, since the amount of electricity in washing is 0.04 kg CO_2_ eq./kg, and the amount of electricity to power the granulator is 0.08 kg of CO_2_ eq./kg, so electricity is negligible for washing and CBP process. The drying process does not have any significant impact. Specifically, the emissions from the drying process amount to 0.13 kg CO_2_ eq./kg, deriving from the only process component, which is electricity, as the drying process takes place in the furnace at a temperature slightly higher room temperature (45 °C).

The results from this study do not represent the environmental impact index of all recycled aggregates, and the range of environmental impact indices must be evaluated through further analyses. However, it is possible to confirm that the cold bonding pelletization process for the production of LWA is a practical and advantageous method in terms of environmental sustainability. 

The application of this analyzed process has widely discussed environmental advantages. This derives from the reduced energy requirement and the reduced formation of secondary pollution, as the granulation process takes place at room temperature and there are no gaseous emissions. This also translates into economic benefits. To this end, an LCC represents a possible future development, to be integrated with the LCA analysis to have a broader view of the advantages brought by the cold bonding pelletization process.

## Figures and Tables

**Table 1:** Chemical composition in percentage by weight (wt%).

Compounds (wt%)	FA	GGBFS	CEM II
Fe_2_O_3_	0.86	25.53	3.41
CaO	24.31	17.48	67.16
CO	16.35	11.29	-
SiO_2_	2.62	-	16.65
Al_2_O_3_	1.53	8.93	4.21
SO_3_	8.57	-	5.34
ClO	21.20	-	-
MgO	1.09	7.94	1.71
ZnO	2.85	-	-
TiO_2_	0.36	-	-
Na_2_O	13.87	-	-
K_2_O	6.41	-	1.54
Mn_2_O_3_	-	3.44	-
Cr_2_O_3_	-	1.84	-
NO_x_	-	10.07	-

**Table 2:** Mixture (wt%) A, B, and C of lightweight aggregates.

Mixture (wt%)	FA	GGBFS	CEM II
LWA A	80	15	5
LWA B	80	10	10
LWA C	80	5	15

**Table 3:** Percentage contribution of main constituents of lightweight aggregates mixtures.

Mixture	FA	GGBFS	CEM II
LWA A	92.73%	6.13%	0.32%
LWA B	94.36%	4.16%	0.66%
LWA C	96.03%	2.12%	1.00%

## Data Availability

Data sharing is not applicable to this article.

## Abbreviations

CBP	Cold Bonding Pelletization
CEM II	Cement (CEM II 42.5) (EN15804 A1-A3)
FA	Fly Ash
FRP	Fiber-reinforced polymer matrix
GGBFS	Ground Granulated Blast Furnace Slag
LCA	Life Cycle Assessment
LCI	Life Cycle Inventory
LCIA	Life Cycle Impact Assessment
LWA	Lightweight Aggregate
LWAC	Lightweight Aggregate Concrete
MSWI	Municipal Solid Waste Incineration
